# Individualized endovascular management of distal posterior circulation aneurysms: a single-center experience and decision framework

**DOI:** 10.3389/fneur.2026.1884020

**Published:** 2026-09-09

**Authors:** Lin Ma, Hao Zhang, Dedi Wu, Jiahang Du, Zhiyong Lu, Huaqiao Tan

**Affiliations:** Department of Interventional Radiology, The Seventh Affiliated Hospital, Sun Yat-Sen University, Shenzhen, China

**Keywords:** anterior inferior cerebellar artery, distal aneurysm, endovascular treatment, posterior cerebral artery, posterior inferior cerebellar artery, superior cerebellar artery

## Abstract

**Purpose:**

Distal intracranial aneurysms (IAs) of the posterior circulation are rare and often pose significant surgical challenges. Although endovascular treatment (EVT) has emerged as an alternative, clear management guidelines remain lacking. We report a single-center experience with EVT for these complex lesions, focusing on procedural outcomes, complications, and follow-up results.

**Methods:**

We retrospectively reviewed patients with distal posterior circulation IAs who underwent EVT at our institution between January 2017 and December 2024. Treatment strategies were individualized based on aneurysm morphology, vascular access tortuosity, parent artery (PA) caliber, distal collateral circulation, and the functional importance of the PA territory. Outcomes assessed included technical success, perioperative complications, angiographic occlusion, and clinical status at the last follow-up, as measured by the modified Rankin Scale (mRS).

**Results:**

Nineteen patients (10 men, 9 women; median age 58 years) with 19 aneurysms were included. Aneurysm locations were posterior cerebral artery (*n* = 8), posterior inferior cerebellar artery (*n* = 7), anterior inferior cerebellar artery (*n* = 3), and superior cerebellar artery (*n* = 1). Fifteen aneurysms were ruptured, and 15 were dissecting in nature. EVT was technically successful in all cases with no intraprocedural complications. Treatment modalities included intra-aneurysmal coiling (*n* = 7), stent-assisted coiling (SAC, *n* = 2), and parent artery occlusion (PAO, *n* = 10). Immediate complete occlusion was achieved in 5/7 coiled aneurysms, 2/2 SAC aneurysms, and all 10 PAO aneurysms. No new neurological deficits occurred in patients who underwent intra-aneurysmal coiling or SAC, while five patients with insufficient collateral circulation who underwent PAO developed territorial infarction with neurological deficits. At a mean follow-up of 8.4 months, all PAO and SAC aneurysms remained stably occluded. However, three of seven coiled aneurysms—all of which were saccular-configured dissecting aneurysms—showed recurrence. No rebleeding occurred. Favorable functional outcomes (mRS ≤ 2) were achieved in 18 patients (94.7%).

**Conclusion:**

For surgically intractable distal posterior circulation aneurysms, EVT represents a viable alternative. Individualized treatment strategies should be formulated according to aneurysm characteristics, vascular access tortuosity, parent artery caliber, distal collateral perfusion, and whether the PA supplies eloquent brain territories. Appropriate individualized management can yield favorable clinical outcomes in the majority of patients.

## Introduction

Distal intracranial aneurysms (IAs) of the posterior circulation arising distal to the origins of the major branches of the vertebrobasilar system are rare lesions that present a unique management challenge (1). Surgical options including direct clip reconstruction, trapping or proximal occlusion with or without distal revascularization, excision with end-to-end anastomosis, or some form of wrapping have been proposed to treat these aneurysms (1–4); however, surgical treatment is still difficult and associated with a rather high morbidity/mortality (5, 6). This is because, in contrast to proximally located IAs, these distal IAs are often small in size and situated deep within the cranial cavity. Moreover, they are frequently adjacent to complex neurovascular structures and involve the circumference of the parent artery (PA) without an apparent neck. Additionally, a significant number of these aneurysms are dissecting aneurysms or pseudoaneurysms, and the fragility of their walls makes them prone to hemorrhage (5, 7). Over the past decade, endovascular treatment (EVT), an attractive minimally invasive treatment modality that spares patients from some of the hazards associated with craniotomy and open surgical clipping, has been widely used to treat distal IAs of the posterior circulation (7–13); however, clear guidelines for their EVT have not been established (1, 14, 15). In the present study, we report our results and experience with EVT for distal IAs of the posterior circulation and discuss their therapeutic strategies.

## Methods

### Patients

This retrospective study was approved by the institutional review board, and patient informed consent was waived due to the retrospective nature of this study. The database of all the patients with distal aneurysm of posterior circulation who underwent EVT was reviewed from January 2017 to December 2024 at our institution. Those patients harboring distal posterior circulation IAs with an obvious underlying cause or known origin, such as infectious, oncotic, or traumatic aneurysms, or those associated with an atrial myxoma, were excluded.

### Strategies of endovascular intervention

Therapeutic alternatives were discussed between the neurosurgical and neuro-interventional teams. Indications for endovascular therapy in our patient group mainly concerned the anticipated surgical difficulties associated with the nature and location of the aneurysm and the overall risk. The selection of EVT strategies was based on multiple factors. These factors included aneurysm characteristics, the degree of tortuosity of the vascular access, the caliber of the PA, and its distal collateral circulation, as well as whether the PA supplies important functional areas.

For saccular aneurysms with a narrow neck or dome-to-neck ratio (D/N) ≥ 2, if the vascular access conditions allow microcatheter access to the aneurysm, intra-aneurysmal coil embolization is the first choice. For aneurysms situated in the proximal segment of the distal vessel, if they are saccular, are wide-necked, or have a D/N < 2, and the PA supplies important functional areas with inadequate collateral circulation in the distal territory, while the diameter of the PA allows the placement of dual low-profile microcatheters and the vascular access can deliver the microcatheters to the target site, stent-assisted coiling (SAC) is the preferred choice. PAO was only adopted for complex distal posterior circulation aneurysms when alternative reconstructive strategies (selective coiling, the balloon remodeling technique, and stent-assisted coiling) were technically infeasible or carried a higher risk of aneurysm rupture, procedural failure, or fatal hemorrhage. Specifically, these lesions were predominantly characterized by (1) complex aneurysm morphologies including wide-necked saccular, fusiform, and dissecting aneurysms with indefinite neck anatomy and fragile walls, which were unsuitable for preservation of the PA; (2) unfavorable vascular conditions, including severely tortuous access vessels and small-caliber parent arteries that precluded reconstructive EVT; and (3) non-eloquent PA territory with adequate potential distal collateral circulation, where permanent parent artery sacrifice would not lead to an irreversible critical neurological deficit.

Preoperative collateral evaluation criteria: Preoperative collateral compensation for PAO in distal posterior circulation aneurysms was systematically evaluated using multimodal imaging. Preoperative CTA and multi-angle DSA were performed to assess anatomical collateral pathways, including vertebral artery symmetry, patency of the distal basilar artery, posterior communicating artery patency, and peripheral vascular anastomoses. Dynamic retrograde filling of the distal vascular territory was evaluated using angiography. Combined with CTP/MRP, we further quantified territorial cerebral perfusion and perfusion reserve to distinguish anatomical and functional collateral compensation. PAO was cautiously considered in patients with sufficient collateral perfusion. For unruptured aneurysms not amenable to coiling or SAC in patients with insufficient collaterals and inadequate collateral reserve, PAO was avoided whenever possible, and detailed patient counseling and shared decision-making were conducted. For emergent ruptured SAH cases without feasible reconstructive strategies, PAO was considered a reasonable alternative treatment option.

**Figure 1:**
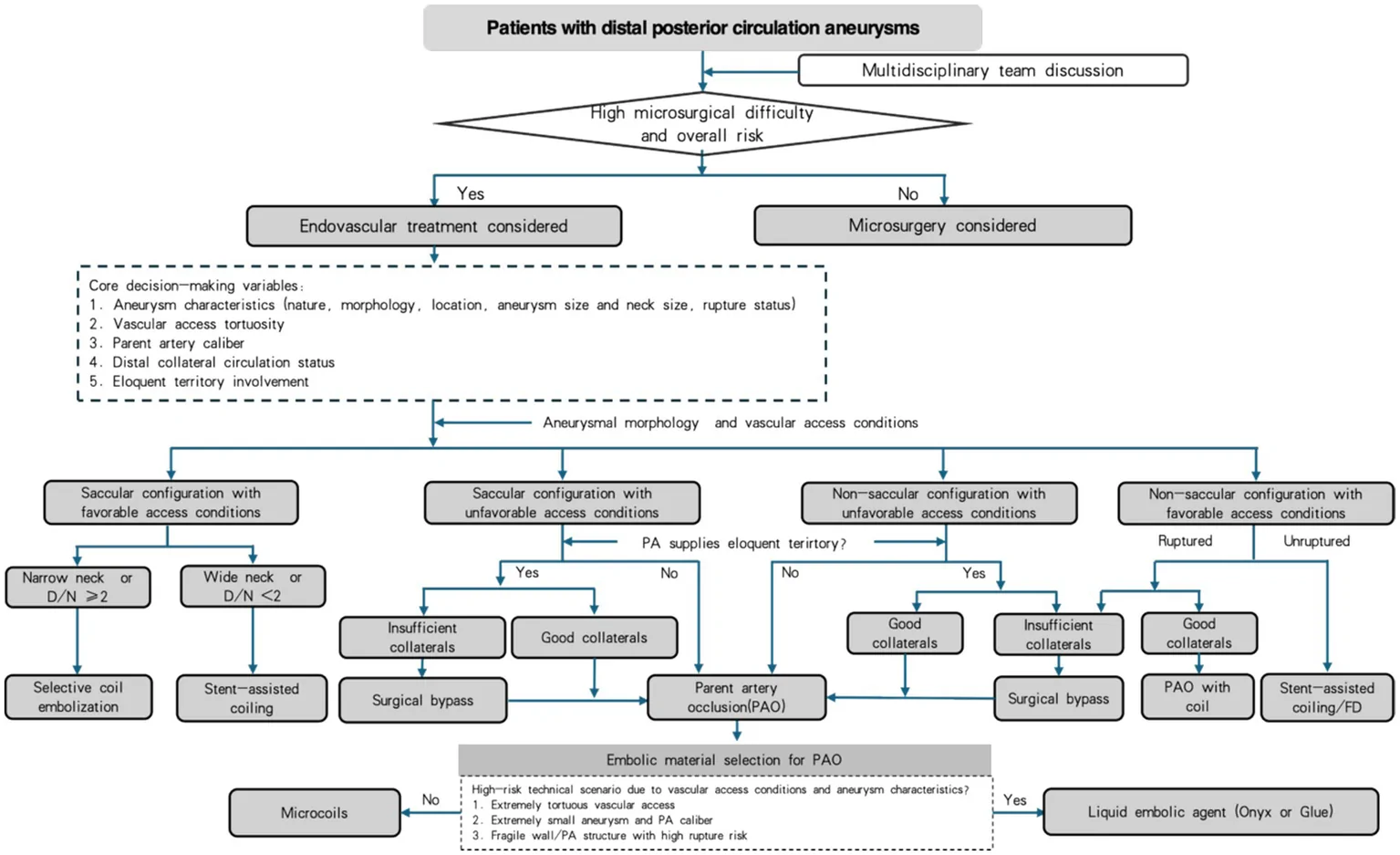
Endovascular treatment algorithm for distal posterior circulation aneurysms. This individualized decision-making framework standardizes therapeutic selection based on five core anatomical and technical variables: Aneurysm morphology, vascular access tortuosity, parent artery caliber, distal collateral circulation status, and eloquent territory perfusion. The hierarchical treatment strategies include selective coil embolization, stent-assisted coiling (SAC), flow diversion (FD), and parent artery occlusion (PAO). For PAO, embolic materials are individually selected according to aneurysm size, pathological features, vascular anatomy, and parent artery diameter to ensure safe and tailored treatment. Favorable vascular access conditions are defined as an adequate vascular trajectory and parent artery diameter that allow superselection microcatheter navigation into the aneurysm cavity and/or permit dual-microcatheter manipulation at the target lesion. EVT, endovascular treatment; D/N, dome-to-neck ratio; PA, parent artery; SAC, stent-assisted coiling; PAO, parent artery occlusion; FD, flow diversion.

The selection of embolic material for PAO depends on the type, location, and size of the aneurysm, the vascular access, and the diameter of the PA. If the vascular access and/or the diameter of the PA do not allow a 0.0165-inch microcatheter to enter the aneurysm cavity, or the size of the aneurysm and the diameter of the PA are too small to occlude with the smallest specification coils, or if the aneurysm wall or PA is too fragile and coil embolization carries a higher risk, the liquid embolic agent is the first choice. The proposed treatment pathway is summarized in Figure 1.

### Endovascular procedure

All procedures were performed under general anesthesia after obtaining informed consent from the patient’s family members. All patients received systemic heparinization to achieve an activated clotting time of approximately 250–300 s during the procedure. For patients who presented with acute SAH, heparinization was started after catheterization. Diagnostic angiography was performed in all patients before embolization. After comprehensive evaluation of aneurysm morphology, vascular access, parent artery diameter, and distal collateral circulation, the endovascular treatment strategy was determined. A 5F or 6F guiding catheter was placed into the proximal vertebral artery at the beginning of the procedure.

In cases of selective coil embolization, the tip of the microcatheter was positioned within the aneurysm sac; subsequently, selective coil embolization was performed in a standard fashion. Coils were selected based on the size of the aneurysm. In cases of SAC, patients with unruptured aneurysms received a dual antiplatelet regimen (clopidogrel, 75 mg/day, and aspirin, 100 mg/day) for at least 3 days before the procedure. In patients with an acute ruptured aneurysm, a loading dose of dual antiplatelet (DAPT) regimen (clopidogrel, 300 mg, and aspirin, 300 mg) was initiated 6 h before the procedure. Whether to use the “coil-through” technique or the “Jailing” technique in SAC was determined by the diameter of the PA, the availability of stent types, and the operator’s preference. Both techniques were performed in a standard fashion, as described in a previous study (16). After treatment, DAPT with aspirin and clopidogrel was continued for at least 6 months. Thereafter, single-antiplatelet therapy was continued for life. In cases of PAO, coils were preferred whenever possible, except in cases of very distal lesions, a small-diameter PA, extreme tortuosity of the vascular access precluding microcatheter navigation to the aneurysm, or excessively fragile aneurysm walls with unacceptably high rupture risk; in these instances, glue was chosen. No balloon test occlusion (BTO) or amobarbital testing was performed prior to PAO in these patients. When performing PAO with coils, the tip of the microcatheter (MTI-ev3, Irvine, CA, USA) for coil delivery was advanced distal to the aneurysmal ostia, and both the parent vessel just proximal and distal to the aneurysm neck and the aneurysm sac were occluded with coils. If the tip of the microcatheter could not be positioned distal to the aneurysm neck, the microcatheter was placed proximally as close as possible to the aneurysmal ostia, and the aneurysm together with the proximal parent vessel anterior to the aneurysm neck was occluded. Coils were selected based on the size of the artery to be occluded. Patients who underwent PAO or occlusion of the aneurysm together with the PA received low-molecular weight heparin calcium 4,000 IU/12 h for the next 3 days. When a liquid embolic agent was used to occlude the PA, the tip of the Marathon microcatheter (Covidien, Irvine, California, USA) or Headway Duo microcatheter (MicroVention, Tustin, California, USA) was navigated to the aneurysmal ostium as close as possible. After confirming the microcatheter tip location by angiogram, under the guidance of a blank roadmap, using the standard technique as described in previous studies (7, 9, 11, 14, 17), Onyx 18 or Glue at the appropriate concentration was injected to occlude the aneurysm and the PA. The injection was terminated when the aneurysm and diseased vessel segment were completely occluded.

After the procedure, angiography was performed immediately to evaluate aneurysm occlusion, and a head CT scan was also obtained for evaluation of possible complications at days 1 and 3. The patient was monitored in the intensive care unit for at least 24 h.

### Follow-up

After the procedure, initial follow-up angiography was performed at 3–6 months. Thereafter, follow-up angiography was decided by the neuro-interventionalist according to the initial follow-up angiographic results. Clinical follow-up was performed to determine neurological deficits at discharge and at every follow-up visit for angiography. More attention was paid to rebleeding of embolized aneurysms and stroke in the territory of the treated artery. Data on initial and final angiographic results and clinical outcomes were retrospectively collected and analyzed by two authors (HQT and LM; by consensus). Clinical outcomes were assessed using the modified Rankin Scale (mRS).

### Definitions

Technical success was defined as successful catheterization and completion of the intended embolization strategy with immediate angiographic exclusion of the aneurysm or the target diseased segment. Complete occlusion was defined as no residual aneurysm filling on immediate or follow-up DSA. Neck remnant or incomplete occlusion was defined as persistent filling of the aneurysm neck or sac. Recurrence was defined as any increase in aneurysm filling or coil compaction on follow-up DSA compared with immediate post-treatment angiography. Complications were categorized as intraprocedural events, including vessel perforation, thromboembolism, parent artery dissection, or aneurysm rupture, and post-procedural treatment-related events, including territorial infarction or new neurological deficits attributable to the treated parent artery. Favorable clinical outcome was defined as mRS ≤ 2 at last follow-up.

### Statistical analysis

Because of the small sample size and descriptive design, no comparative hypothesis testing was performed. Continuous variables are presented as medians with ranges or means +/− standard deviations, as appropriate. Categorical variables are presented as counts and percentages.

## Results

### Baseline characteristics of patients and aneurysms

Nineteen patients who presented to our institution with distal IAs of the posterior circulation and were treated endovascularly were included in this study. The characteristics of the patients and the aneurysms are summarized in Table 1. There were 10 male patients and 9 female patients with a median age of 58 years (range, 26 to 69 years). One patient had an incidentally found aneurysm related to hemodynamics due to an arteriovenous malformation (AVM). As a result of aneurysm rupture, 1 patient presented with intracerebral hemorrhage and SAH, 1 patient presented with intraventricular hemorrhage and SAH, and 13 patients presented with SAH alone. In 3 patients, the aneurysm was asymptomatic and incidentally found. According to the Hunt–Hess scale, 4 patients were grade 0, 1 patient was grade 1, 11 patients were grade 2, 2 patients were grade 3, and 1 patient was grade 4.

**Table 1:** Baseline characteristics of 19 patients with distal posterior circulation aneurysms.

Characteristic	No. (%)
Sex	
Male	10 (52.6%)
Female	9 (47.4%)
Age, median, y	58 (26–69)
Aneurysm location	
PCA	8 (42.1%)
PICA	7 (36.8%)
AICA	3 (15.8%)
SCA	1 (5.3%)
Pathological type	
True saccular aneurysm	4 (21.1%)
Dissecting aneurysm	15 (78.9%)
Aneurysm morphology	
Saccular	14 (73.7%)
Fusiform	2 (10.5%)
Irregular	3 (15.8%)
Clinical presentation	
Aneurysm ruptured	15 (78.9%)
SAH alone	13 (68.4%)
SAH + intracerebral hemorrhage	1 (5.3%)
SAH + intraventricular hemorrhage	1 (5.3%)
Aneurysm unruptured	4 (21.1%)
Hunt-Hess grade	
0	4 (21.1%)
1	1 (5.3%)
2	11 (57.9%)
3	2 (10.5%)
4	1 (5.3%)

IA, intracranial aneurysm; PCA, posterior cerebral artery; PICA, posterior inferior cerebellar artery; AICA, anterior inferior cerebellar artery; SCA, superior cerebellar artery; SAH, subarachnoid hemorrhage.

The locations of the aneurysm were as follows: posterior cerebral artery (PCA) in eight patients, anterior pontomesencephalic segment of the superior cerebellar artery (SCA) in one patient, lateral pontine segment of the anterior inferior cerebellar artery (AICA) in three patients, and posterior inferior cerebellar artery (PICA) in seven patients. Of the seven PICA aneurysms, four arose from the lateral medullary segment, one from the cortical segment, and two from the telovelotonsillar segment. Of the eight PCA aneurysms, seven were located at the P2 segment and one at the P1–P2 junction. Of the three AICA aneurysms, two arose from the meatal segment and the remaining one at the premeatal segment.

Among the 19 aneurysms, 4 were considered to be true saccular aneurysms, and the remaining 15 were dissecting aneurysms. All aneurysms were small, except for two large dissecting aneurysms. Of the 4 true aneurysms, all were narrow-necked (< 4 mm) with a D/N < 2 in 2 patients and a D/N ≥ 2 in 2 patients. Of the 15 dissecting aneurysms, 10 were saccular-configured, 2 were fusiform, and 3 were irregular in shape; 5 were without a definite neck, and the remaining 10 were narrow-necked, with a D/N < 2 in 8 patients and a D/N ≥ 2 in 2 patients.

### Immediate angiographic results

EVT was technically successful in all cases. Table 2 shows the summary of treatment, outcome, and follow-up data. During the operation, no procedure-related complications occurred, including vessel perforation, thrombosis, and aneurysm rupture. Detailed case-by-case clinical characteristics, aneurysm features, treatment strategies, and follow-up outcomes are provided in Supplementary Table 1.

**Table 2:** Summary of treatment, outcome, and follow-up data.

Variable	No. (%)
Technical success	19 (100%)
Treatment strategy	
Coil embolization	7 (36.8%)
SAC	2 (10.5%)
PAO with coil	4 (21.1%)
PAO with Onyx	4 (21.1%)
PAO with Glubran glue	1 (5.3%)
PAO with coil plus glue	1 (5.3%)
Immediate embolization outcome	
Complete occlusion (Raymond I)	17 (89.5%)
Neck remnant (Raymond II)	1 (5.3%)
Incomplete occlusion (Raymond III)	1 (5.3%)
Complications	
Intraprocedural complications	0
PAO-related territorial infarction/new neurological deficit	5 (26.3%)
Rebleeding	0
Follow-up	
Mean follow-up time, mean ± SD, months	8.37 ± 2.56
Imaging follow-up with DSA	19 (100%)
Stable complete occlusion at follow-up DSA	16 (84.2%)
Recurrence on follow-up DSA	3 (15.8%)
Clinical follow-up	
Rebleeding during follow-up	0
mRS score at last follow-up, mean ± SD	0.53 ± 1.02
mRS 0	13 (68.4%)
mRS 1	4 (21.1%)
mRS 2	1 (5.3%)
mRS 3	0
mRS 4	1 (5.3%)
Favorable outcome (mRS ≤ 2)	18 (94.7%)

SAC, stent-assisted coil embolization; PAO, parent artery occlusion; DSA, digital subtraction angiography; mRS, modified Rankin Scale; SD, standard deviation.

#### Selective coil embolization

**Figure 2:**
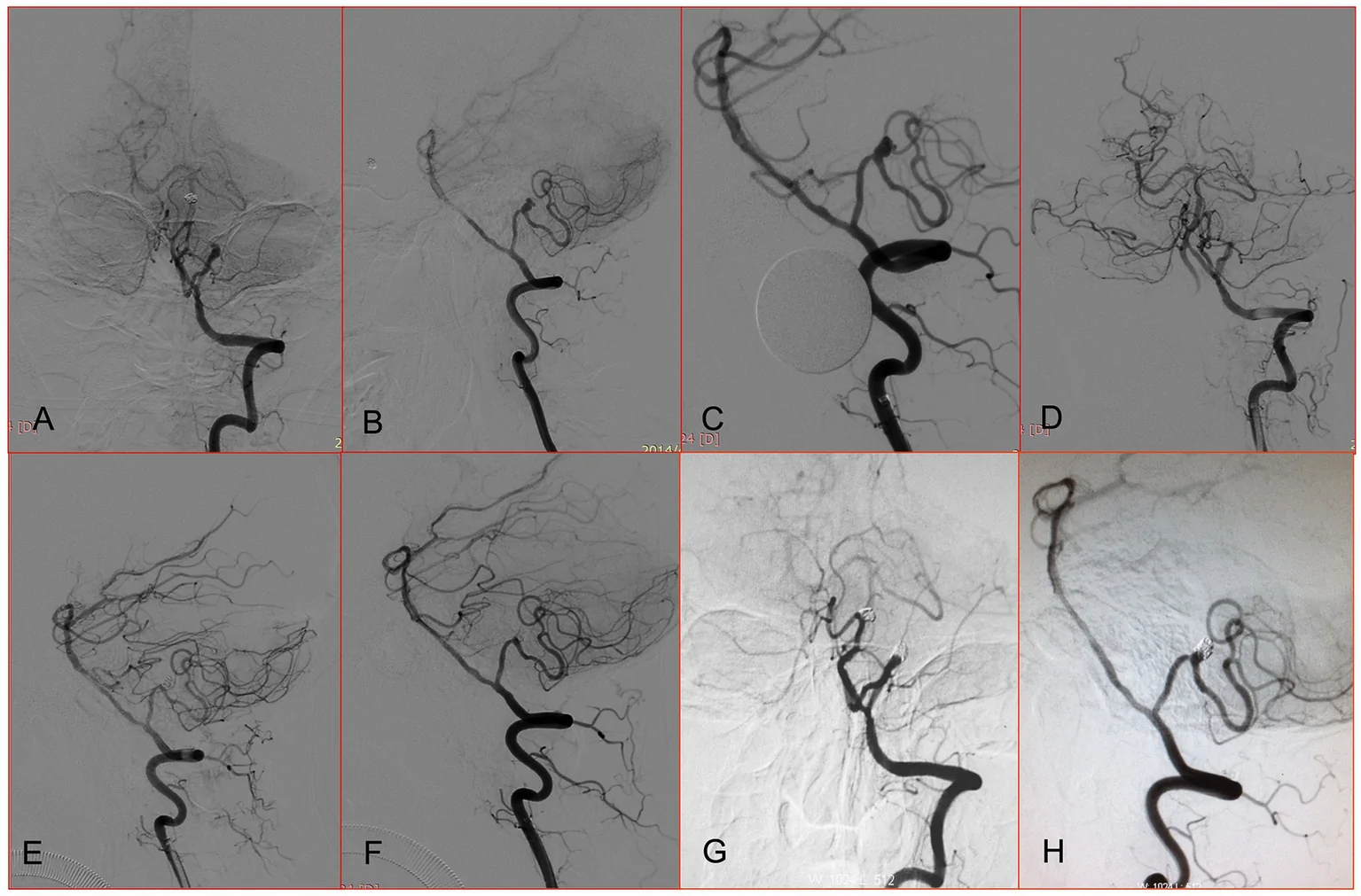
Case 3. 60-year-old female patient presented with sudden-onset severe headache and was diagnosed with subarachnoid hemorrhage (SAH) due to rupture of a posterior inferior cerebellar artery (PICA) aneurysm. She had a history of prior SAH resulting from rupture of an anterior communicating artery aneurysm. **(A–C)** Left vertebral artery angiograms [anteroposterior **(A)**, lateral **(B)**, and oblique **(C)**] demonstrate a small saccular-configured dissecting aneurysm (3.4 × 2.6 mm, neck 2 mm) at the lateral medullary segment of the PICA. **(D–F)** Immediate angiogram of left vertebral artery [anteroposterior **(D)**, lateral **(E)**, and oblique **(F)**] after intra-aneurysmal coil embolization shows complete aneurysm occlusion with preserved patency of the PICA. **(G,H)** Follow-up angiograms at 9 months post-procedure [anteroposterior **(G)** and lateral **(H)**] reveal a residual aneurysm neck consistent with aneurysm recurrence and coil compaction.

**Figure 3:**
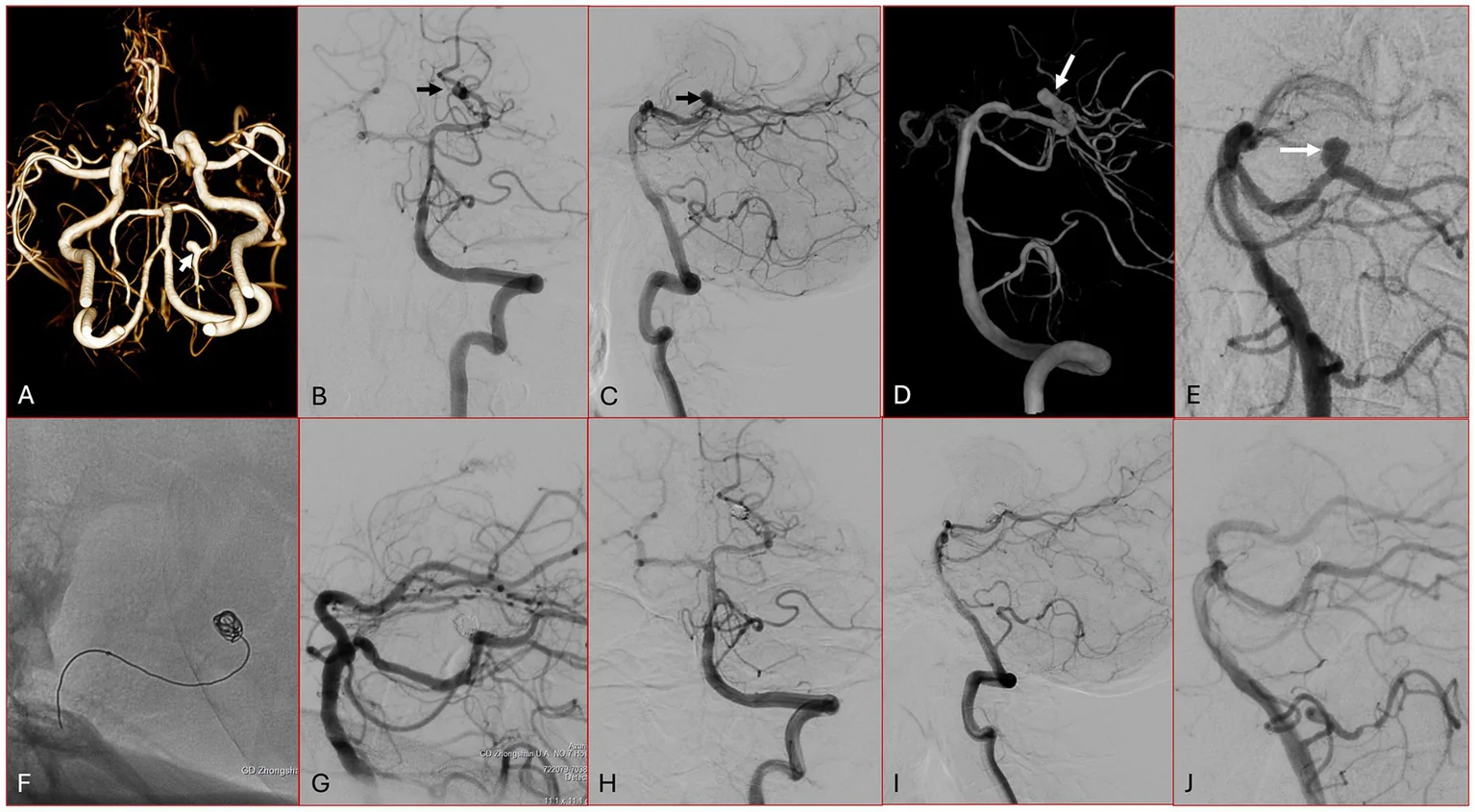
Case 4. 69-year-old asymptomatic male patient with an incidentally detected aneurysm. **(A)** Head CTA demonstrates a 5 × 4 mm saccular aneurysm (white arrow) with a 2.5-mm neck, located at the P2P segment of the left posterior cerebral artery (PCA). **(B,C)** Left vertebral artery angiograms [anteroposterior **(B)** and lateral **(C)**] demonstrate a saccular aneurysm (black arrow) at the P2P segment of the left PCA. **(D,E)** Volume-rendered (VR) reconstruction of 3D-DSA **(D)** and working view of left vertebral artery angiogram **(E)** demonstrate a saccular aneurysm (white arrow) at the P2P segment of the left posterior cerebral artery (PCA). **(F)** X-ray spot film demonstrates coil embolization of the aneurysm following 3D framing coil deployment. **(G)** Immediate post-procedural left vertebral artery angiogram shows complete occlusion of the aneurysm. **(H–J)** Follow-up angiograms at 12 months post-procedure [anteroposterior **(H)**, lateral **(I)**, and tangential **(J)**] demonstrate stable complete aneurysm occlusion.

Seven patients underwent intra-aneurysmal coil embolization. Representative cases are shown in Figures 2, 3. Of these, four had true saccular aneurysms, and three had saccular-configured dissecting aneurysms. Immediate complete angiographic occlusion was accomplished in all four true saccular aneurysms after the procedure (Figure 2). Of the three saccular-configured dissecting aneurysms, one achieved complete occlusion (Figure 3), one had a neck remnant, and one had incomplete occlusion.

#### Stent-assisted coiling

Two patients with saccular-type dissecting aneurysms were treated with SAC. One aneurysm was located at the lateral medullary segment of the PICA and the other at the P1-2 junction of the PCA. Both lesions had a D/N < 2. No procedure-related complications occurred, including vessel perforation, aneurysm rupture, or in-stent thrombosis. Immediate complete occlusion with patency of the PA was achieved in both patients.

#### Parent artery occlusion

**Figure 4:**
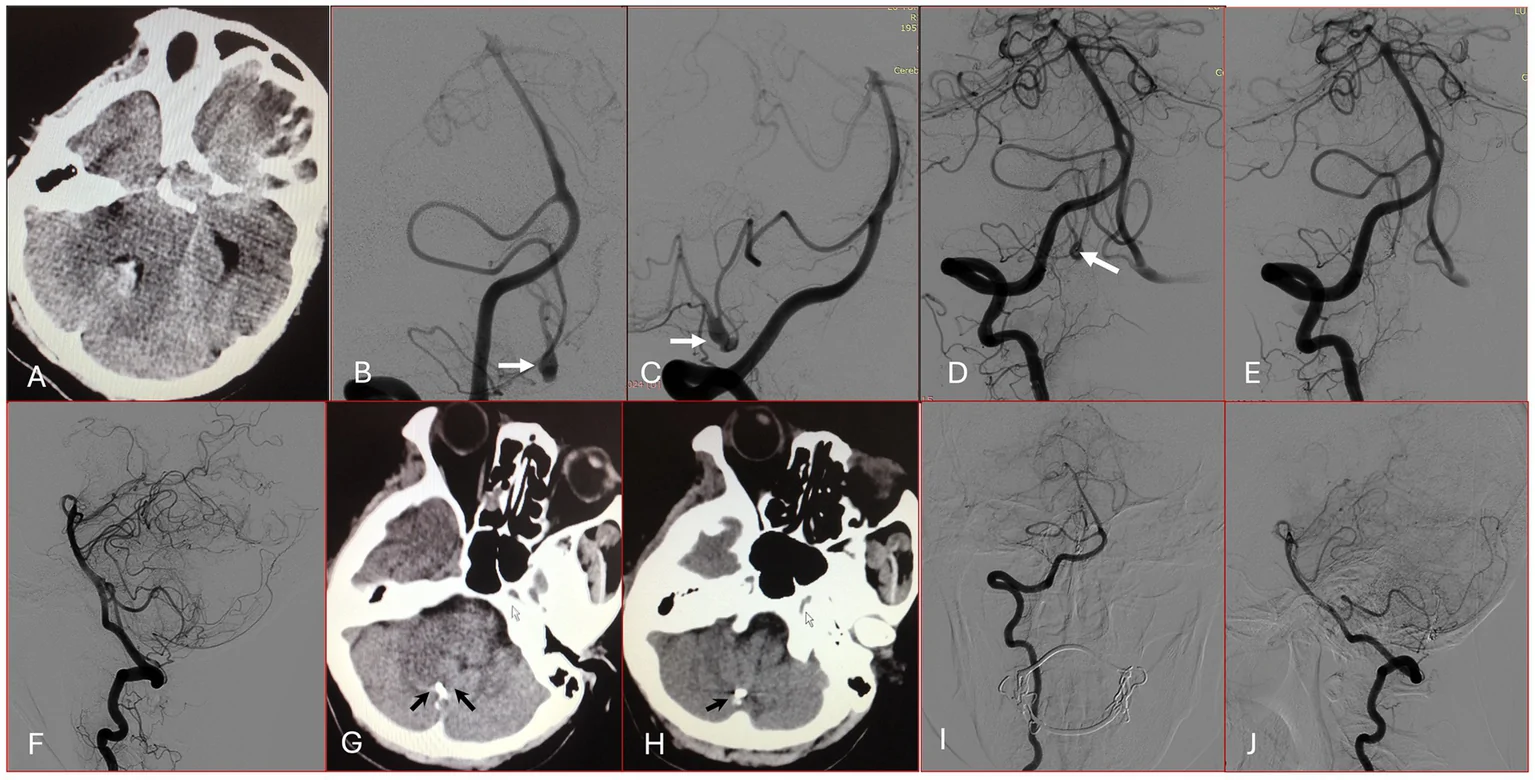
Case 16. 57-year-old female patient presented with sudden-onset headache; head computed tomography (CT) demonstrated subarachnoid hemorrhage (SAH). **(A)** Head CT demonstrates subarachnoid hemorrhage (SAH) and hemorrhage within the fourth ventricle, accompanied by mild hydrocephalus. **(B,C)** Right vertebral artery angiograms [anteroposterior **(B)** and right oblique **(C)**] show a 7 × 2.5 mm saccular-configured dissecting aneurysm (white arrow) with a daughter sac and a 2.5-mm neck, located at the cortical segment of the posterior inferior cerebellar artery (PICA), with an extremely tortuous vascular access. **(D)** The tip of the Marathon microcatheter (white arrow) was positioned within the aneurysm sac. **(E,F)** Right vertebral artery angiograms [anteroposterior **(E)** and lateral **(F)**] obtained immediately after parent artery occlusion (PAO) with Onyx-18 show complete occlusion of the aneurysm and the adjacent parent artery, with robust collateral circulation between the proximal and distal branches of the occluded parent artery. **(G,H)** Head CT obtained on post-procedural day 3 shows no infarction in the PICA territory and Onyx deposition within the lesion (black arrow). **(I,J)** Follow-up angiograms at 12 months post-procedure [anteroposterior **(I)** and lateral **(J)**] demonstrate stable complete aneurysm occlusion.

Ten patients were treated with PAO: nine underwent occlusion of the aneurysm together with the PA, and one received proximal parent artery occlusion anterior to the aneurysm ostium. Four patients were embolized with coils, four with Onyx, one with glue, and one with a coil-glue combination. A representative case is shown in Figure 4. Complete aneurysm occlusion was achieved in all 10 patients immediately after the procedure. Retrograde filling of the distal branches of the occluded artery via leptomeningeal collaterals was observed in four patients. In the patient who underwent proximal PAO, the retrograde filling of distal branches was noted, but no retrograde flow into the aneurysm was identified. The remaining five patients showed no retrograde filling of the distal branches of the occluded artery.

### Initial clinical results

After the procedure, all patients survived. None of the patients who underwent intra-aneurysmal coil embolization or SAC developed neurologic deficits, and post-procedural CT scans showed no infarction in the territory of the PA. Among the 10 patients who underwent PAO, five patients with adequate collateral circulation had an uneventful course, which was confirmed on post-procedural CT. The remaining five patients who lacked collateral circulation in the distal vessel of the occluded PA developed anticipated neurological deficits. Three patients (cases 2, 10, and 14) with an aneurysm located at the P2 segment of the PCA developed homonymous hemianopsia and weakness and numbness of the contralateral limb 24 h after treatment. Post-procedural CT at day 3 revealed a small thalamic infarction and medial temporal lobe and occipital lobe infarctions. One patient (case 13) with an aneurysm located in the lateral medullary segment of the PICA developed Wallenberg syndrome, presenting with dysphagia, water-swallowing aspiration, paresthesia, and limb ataxia. Post-procedural CT at day 3 revealed a left cerebellar and medulla infarction. One patient (case 11) with an aneurysm located in the meatal segment of the AICA developed ipsilateral cerebellar ataxia and diminished or absent ipsilateral facial and contralateral body superficial sensations. Post-procedural CT at day 3 revealed infarction in the territory of the AICA.

At the time of discharge, an mRS assessment showed mRS 0 in 10 patients, mRS 1 in five patients, mRS 2 in two patients, mRS 3 in one patient, and mRS 5 in one patient. Two patients (cases 10 and 13) with mRS ≥ 3 had unfavorable outcomes attributable to the severity of their initial hemorrhage.

### Follow-up angiographic results

All patients underwent a follow-up angiogram 6–12 months after treatment; the mean follow-up period was 8.37 ± 2.56 months. In the 10 patients who underwent PAO, complete aneurysm occlusion remained stable. Among the two patients who underwent SAC, complete aneurysm occlusion remained stable, while stent patency was maintained. Among the seven patients treated with intra-aneurysmal coil embolization, four patients with true saccular aneurysms maintained stable complete aneurysm occlusion. Aneurysm recurrence developed in three patients with saccular-configured dissecting aneurysms: One of these patients had initial complete aneurysm occlusion, while the remaining two had initial neck remnants or incomplete aneurysm occlusion. These recurrent aneurysms were subsequently retreated with either selective coil embolization or SAC.

### Follow-up clinical results

All patients underwent clinical follow-up for 6–12 months, and the mean follow-up period was 8.37 ± 2.56 months. None of the patients had an episode of rebleeding after treatment. At last follow-up, the mRS assessment showed mRS 0 in 13 patients, mRS 1 in four patients, mRS 2 in one patient, and mRS 4 in one patient. Compared with the initial mRS at the time of discharge, the mRS in 12 patients remained stable, and the mRS in seven patients showed obvious improvement. Overall, 18 patients had favorable functional outcomes (mRS ≤ 2). One patient had a permanent severe neurologic deficit. One patient (case 10) with a PCA aneurysm had persistent paralysis of the contralateral limb and homonymous hemianopsia.

## Discussion

In the present study, distal IAs of the posterior circulation were managed with individualized EVT strategies, based on aneurysm characteristics, vascular access tortuosity, caliber of the PA, distal collateral circulation status, and whether the PA supplies important functional areas. Our findings indicate that EVT represents a viable alternative for surgically intractable distal posterior circulation aneurysms. Through meticulous and individualized management, the vast majority of patients achieved favorable outcomes. The individualized treatment strategies for distal IAs in the posterior circulation proposed herein may provide a valuable reference for clinicians in formulating treatment decisions.

### Selection of endovascular treatment modality for distal IAs of the posterior circulation

Currently, numerous EVT modalities have been proposed for distal posterior circulation IAs, including PAO with coils or liquid embolic agents (6, 7, 9, 18–23), intra-aneurysmal embolization (5, 20), balloon- or stent-assisted coiling (SAC) (24), stent monotherapy (25), and flow diverter (FD) therapy (26–32). The optimal EVT approach is to occlude the aneurysm while preserving the patency of the PA. Intra-aneurysmal embolization represents the optimal therapeutic strategy; however, not all distal posterior circulation IAs are amenable to this approach. Intra-aneurysmal embolization is only suitable for saccular aneurysms with a relatively narrow neck and favorable vascular access that allows microcatheter navigation into the aneurysm sac. Reconstructive treatment techniques, such as SAC or balloon remodeling techniques, can occlude the aneurysm while preserving PA patency but frequently require a PA diameter sufficient to accommodate dual-microcatheter manipulation. This makes them suitable only for aneurysms located in the proximal segment of distal vessels, where the vascular access permits the delivery of two microcatheters to the target site. For those aneurysms not amenable to intra-aneurysmal embolization or reconstructive treatment, PAO has been advocated as a viable alternative (18–20, 33, 34).

In our institution, we have developed a treatment algorithm for distal posterior circulation IAs based on aneurysm characteristics, vascular access tortuosity, parent artery caliber, distal collateral circulation status, and whether the PA supplies important functional areas. According to this treatment algorithm, seven patients were treated with intra-aneurysmal coil embolization; complete aneurysm occlusion was achieved in five patients, neck remnant in one patient, and incomplete occlusion in one patient. Two patients underwent SAC, with complete aneurysm occlusion and preservation of PA patency achieved in both cases. Ten patients were treated with PAO, and immediate complete aneurysm occlusion was achieved in all these patients. EVT was technically successful in all cases, and no intraprocedural complications occurred. Notably, five of 10 patients who underwent PAO developed territorial infarction and neurological deficits because of the insufficient collateral circulation. No patient experienced rebleeding after treatment. In patients who underwent PAO or SAC, follow-up angiography demonstrated stable complete aneurysm occlusion. Among the seven patients who underwent intra-aneurysmal coil embolization, follow-up angiography showed stable complete occlusion in four patients with true saccular aneurysms, while aneurysm recurrence was observed in three patients harboring saccular-configured dissecting aneurysms. At the last follow-up, 18 patients had favorable functional outcomes (mRS ≤ 2), and one patient had a permanent severe neurological deficit. Our results are comparable to those reported in previous studies on EVT for distal posterior circulation IAs (8, 20, 35–44). These findings support the conclusion of prior studies that the EVT modality for distal posterior circulation IAs should be individualized based on the intrinsic characteristics of the aneurysm, vascular anatomy, whether the PA supplies the functional area, and its collateral circulation (8, 37, 45–47).

### Selection of embolic agent for intra-aneurysmal embolization

Although intra-aneurysmal embolization with Onyx has been reported to be a potentially effective option for the treatment of distal cerebellar aneurysms that cannot be managed by selective coil occlusion of the aneurysmal sac or open craniotomy clipping (5, 48), seven patients with distal posterior circulation IAs eligible for intra-aneurysmal embolization underwent coil embolization in this study. In our experience, coils are the most widely used embolic materials for intra-aneurysmal embolization; intra-aneurysmal coil embolization is safer and more controllable. First, intra-aneurysmal embolization with Onyx demands highly proficient techniques and extensive experience (5), which are not universally available among neuro-interventionalists. Second, Onyx embolization of aneurysms with PA preservation is extremely challenging because the small caliber of the PA precludes the use of a balloon to seal the aneurysm, a critical step in Onyx embolization for IAs (49). Third, intra-aneurysmal embolization with Onyx does not reliably achieve durable aneurysm occlusion while maintaining PA patency and carries a risk of proximal reflux or distal migration of Onyx. In the series reported by Gao et al. (5), among the 10 patients with distal cerebellar aneurysms treated with Onyx, two patients developed silent cerebellar infarcts and one experienced inevitable vessel occlusion. This makes them only suitable for selected patients with specific aneurysm characteristics.

### Selection of techniques and embolic materials for PAO

For distal posterior circulation IAs that are difficult or even impossible to treat with intra-aneurysmal coiling or reconstructive techniques, PAO using either coils or liquid embolic agents (e.g., Onyx or glue [Glubran/n-butyl cyanoacrylate]) has been recognized as a viable alternative treatment option (6, 7, 9, 11, 14, 15, 17, 18, 20, 50). Coil embolization was the most widely used treatment modality. Compared with liquid embolic agents, coils are more maneuverable, enabling more precise placement without the risk of distal migration or proximal reflux of liquid embolic agents. This technique only occludes the aneurysm and the PA adjacent to the aneurysm neck, while the proximal portion of the PA and the perforating arteries originating from these occluded segments remain patent. Distal branches of the occluded artery can obtain retrograde perfusion through leptomeningeal collateral circulation, thereby minimizing the risk of symptomatic ischemic complications (20). In addition, there is no risk of microcatheter entrapment or adhesion during coil embolization. However, the use of coils for PAO or combined aneurysm and PA occlusion has some inherent limitations. This technique requires the tip of the microcatheter to be able to enter the aneurysm or advance as close to the aneurysm as possible for coil delivery. For aneurysms located in extremely distal vessels, or those with excessively tortuous vascular access and small-caliber PAs, super-selective catheterization may be difficult or even impossible, accompanied by a relatively high risk of aneurysm rupture and PA perforation (9). In addition, for dissecting aneurysms located in extremely distal vessels, intra-aneurysmal coiling may increase the risk of rupture due to the interaction between the coils and the fragile aneurysm wall (9).

In such cases, PAO using liquid embolic agents may be an appropriate alternative. Unlike microcatheters for delivering coils, flow-directed microcatheters for injecting liquid embolic agents (e.g., Marathon or Headway Duo microcatheters) are softer and can be easily navigated to extremely distal vessels through excessively tortuous vascular access (5, 14). Furthermore, even if the microcatheter cannot reach the aneurysm sac, the liquid embolic agent can diffuse into the aneurysm sac due to the excellent diffusion of both Glubran glue and Onyx, avoiding the risk of rebleeding under the occlusion of PA (15). Meanwhile, compared with coil embolization, PAO with liquid embolic agents has a lower risk of aneurysm rupture because no manipulation of coils is required (5, 7). However, PAO with liquid embolic agents is not without drawbacks. Despite the adoption of high-quality fluoroscopy, the “reflux-hold-reinjection” technique, and intermittent control angiographies, this technique still carries the potential risk of distal migration or proximal reflux of the embolic agent, which may occlude potential collateral vessels (5). In addition, liquid embolic agents may also pose a potential risk of microcatheter entrapment or adhesion (5, 15).

In this study, we preferred coils as the first choice for PAO or occlusion of the aneurysm together with the PA; however, for aneurysms located in extremely distal vessels, those with excessively tortuous vascular access and small-caliber PAs (where super-selective catheterization may be difficult or even impossible), or for distally located dissecting aneurysms with fragile walls, liquid embolic agents were selected for occlusion of the aneurysm together with the PA. In our series, when considering the use of liquid embolic agents for PAO, Onyx was preferred as the first choice. This is because Onyx is a non-adhesive liquid embolic agent that is visualizable and easier to handle. Compared with n-butyl cyanoacrylate/Glubran, Onyx allows for more prolonged, slow, and controlled injection, thereby increasing the rate of PA preservation, reducing the risk of distal migration or proximal reflux of the embolic agent, and thus minimizing the risk of symptomatic ischemic complications (5, 7, 9). Moreover, Onyx embolization decreases the risk of microcatheter entrapment or adhesion of the catheter to the vessel wall (5, 7, 15). However, this technique also has some disadvantages, for example, increased fluoroscopy time and potential adverse effects of dimethyl sulfoxide (5).

In this study, 10 patients underwent PAO: Nine underwent occlusion of the aneurysm together with the PA, and one underwent proximal PAO anterior to the aneurysm ostium. Immediate complete aneurysm occlusion was achieved in all 10 patients, and no intraprocedural complications occurred. Despite the high technical success rate of endovascular PAO for distal posterior circulation aneurysms, this procedure is associated with non-negligible ischemic complications. In this study, five patients who underwent PAO developed postoperative territorial infarction and subsequent neurological deficits. These neurological deficits were primarily attributed to focal cerebral hypoperfusion and insufficient collateral circulation compensation after parent artery sacrifice. Therefore, extreme caution must be exercised when considering PAO for the treatment of distal posterior circulation aneurysms that are not suitable for selective coil embolization, remodeling technique, or SAC, and strict preoperative assessment of collateral circulation and eloquent territory perfusion should be performed. For individuals with inadequate collateral perfusion or functional territory dependence, surgical revascularization should be considered prior to PAO to reduce postoperative ischemic morbidity. For patients with high-risk, surgically and endovascularly intractable aneurysms, PAO was adopted as a salvage therapeutic strategy after thorough risk–benefit evaluation.

### Consideration of BTO or amytal testing before PAO for distal posterior circulation IAs

The potential risk of PAO or occlusion of the aneurysm combined with the PA is ischemia in the territory supplied by the occluded vessel (5, 15, 18, 20, 37, 38, 51). Although BTO or amobarbital testing has been proposed to predict the neurological deficits that the patient may experience after PA occlusion (5, 9, 14, 18, 19, 50), the results of these tests are notoriously unreliable (6, 18, 19, 37), and there is currently no test that can definitively assess the adequacy of collateral circulation (1, 11). In this study, BTO or amobarbital testing was not performed before PAO because these patients were in the acute stage of SAH and were unable to cooperate with amobarbital testing, and selective BTO was technically difficult because of the small caliber of the distal PA. Additionally, these tests are not considered critical for treatment planning, as an amobarbital test or BTO may yield false-positive or false-negative results, and in many cases, the treatment plan remains unchanged even if the patient cannot tolerate the test (5, 20). Based on our experience, PAO or occlusion aneurysm combined with the PA is an acceptable alternative in exceptional cases. However, we emphasize that PAO or aneurysm occlusion combined with PA occlusion should only be considered for distal posterior circulation IAs that are difficult or impossible to treat with intra-aneurysmal embolization or reconstructive technique.

### Future directions for the treatment of distal posterior circulation IAs

In the past decade, FDs have been used off-label for the treatment of distal IAs beyond the circle of Willis. Although numerous studies have shown that FDs are feasible and effective in the treatment of distal IAs with relatively high complete occlusion rates (26–28, 52–54), their application in distal IAs remains controversial and technically challenging. Distal intracranial vessels are often less than 2 mm in diameter with extremely tortuous access. The delivery of conventional FDs requires microcatheters with an inner diameter of 0.021–0.027 inches; these microcatheters are large in caliber and inherently relatively stiff, making it extremely difficult to navigate and cannulate them into small-sized, increasingly tortuous distal intracranial vessels (28), and this maneuver is also likely to increase the risk of vascular perforation (29). In addition, due to the small caliber of the PA and the relative stiffness of high-metal-coverage stents, the deployment of FDs in the distal intracranial vessel is technically challenging (28). Therefore, the off-label use of conventional FDs for the treatment of distal IAs must be strictly based on individual vascular anatomy.

In recent years, novel low-profile FDs (such as SILK Vista Baby, Balt; FRED Jr., MicroVention) designed for distal IAs with excellent navigability have been approved for use in small, distal intracranial vessels, and several studies have confirmed the safety and efficacy of such low-profile FDs in the treatment of distal IAs (29–32, 55). However, no FDs were used in this study for the treatment of distal posterior circulation IAs; instead, classic EVT modality was adopted, including intra-aneurysmal coil embolization, SAC, or PAO, for the following main reasons: First, novel low-profile FDs have only been approved for marketing in recent years and are not available at our institution. Second, complications associated with FD deployment are non-negligible, including ischemic/thromboembolic and hemorrhagic complications (26, 30, 31, 53). According to reports, in series studies on the treatment of distal IAs with FDs, the overall incidence of complications is relatively high, ranging between 15 and 20%, of which approximately 10% are permanent neurologic deficits (53). Third, distal IAs are often associated with branch vessels and perforators, and in many cases, branch vessels arise directly from the aneurysms. When treating such aneurysms with FDs, the relevant branch vessels and perforators originating near the aneurysm or incorporating into its neck would inevitably be jailed by the endoluminal reconstruction device. The fate of these jailed branch vessels and perforators remains an important concern. It has been reported that after FD implantation, the jailed branch vessel may develop narrowing in 10–20% of cases (26), complete occlusion in 0–20% (26, 31), and jailed perforator occlusion in 1.8–10.7% (26, 32). In the future, with the popularization of FDs specifically designed for the treatment of distal IAs, FDs may be considered as a treatment option for some distal posterior circulation IAs that other available treatment modalities are either technically unfeasible or have a higher overall risk. However, we emphasize that strict patient selection is still required for the treatment of distal posterior circulation IAs with FDs. FDs may be suitable for distal posterior circulation IAs that are located relatively proximally and have a relatively larger caliber, favorable vascular access conditions, and no acute angulation of the PA.

### Study limitations

This study has several limitations. First, it was a retrospective single-center study with a very small sample size of 19 patients, limiting statistical power, subgroup analysis, and generalizability. Second, the heterogeneity in aneurysm characteristics, including variable nature, morphology, size, and anatomic location across our cohort, represents both an important limitation and a critical factor when interpreting the present findings. Distal posterior circulation lesions encompass a wide spectrum of configurations, including different aneurysm sizes, neck morphologies, and varying degrees of vascular tortuosity, all of which substantially influence technical difficulty, device deployment behavior, hemodynamic modification, and final angiographic obliteration. The inherent heterogeneity of this patient cohort inevitably limits direct and standardized comparisons among different endovascular treatment modalities. Therefore, future studies should strive to establish more homogeneous subgroups stratified by aneurysm location, morphology, and angioarchitecture. Further standardized and stratified analyses will enable more rigorous, reliable comparisons of different EVT strategies and help define optimal treatment indications for distinct subtypes of distal posterior circulation intracranial aneurysms. Third, there was no surgical or conservative control group, and treatment selection was not randomized, but based on multidisciplinary clinical judgment, introducing selection bias. Fourth, because of the observational design and small cohort, we could not control for important confounders such as aneurysm morphology, parent artery caliber, collateral status, operator experience, institutional volume, and device availability. Fifth, publication bias in the literature may inflate the perceived safety and efficacy of EVT for rare distal aneurysms because favorable case series are more likely to be reported. Sixth, angiographic and clinical follow-up were relatively short, and longer follow-up is required, especially after selective coiling and SAC. However, our results are consistent with those from other institutions and align with the existing literature. Therefore, the proposed decision framework should be considered hypothesis-generating and experience-based rather than externally validated.

## Conclusion

For distal posterior circulation IAs that are intractable surgically, EVT represents a viable alternative. Individualized treatment strategies should be formulated according to aneurysm characteristics, vascular access tortuosity, parent artery caliber, distal collateral perfusion, and whether the PA supplies eloquent brain territories. Appropriate individualized management yields favorable clinical outcomes in most patients.

## Data Availability

The original contributions presented in the study are included in the article/Supplementary material, further inquiries can be directed to the corresponding author.
