# Supplementary material for: Individualized endovascular management of distal posterior circulation aneurysms: a single-center experience and decision framework
Source: Front Neurol. 2026 Sep 9;17:1884020. doi: 10.3389/fneur.2026.1884020 (PMC13598280; doi:10.3389/fneur.2026.1884020)
Supplement: Supplementary file 1 [file Table_1.pdf]

Table 1 Clinical and Aneurysm Characteristics, and Endovascular Treatment Outcomes in 19 Patients with Distal Posterior Circulation Aneurysms

Case No.	Sex/ Age	Clinical presentations	HHS	Aneurysm characteristics				Treatment strategy	Immediate angiographic results	Procedural complications	Discharge (mRS)	Follow-up		
				Site	Pathological type/ morphology	Size (mm)	Neck (mm)					Duration (m)	DSA	mRS
1	M/40	Headache, AVM	0	L PCA: P1-2 junction	True/ Saccular	4	2	Coiling	Complete occlusion	No	0	12	Complete occlusion	0
2	F/53	SAH	2	L PCA: P2	Dissecting/ Saccular	4.6	3.1	PAO;coil	Complete occlusion	CI:temporal occipital lobe	2	6	Complete occlusion	1
3	F/60	SAH	2	L PICA: lateral medullary	Dissecting/ Saccular	3.4	2	Coiling	Complete occlusion	No	0	9	Recurrence	0
4	M/69	Asymptomatic	0	L PCA, P2P	True/ Saccular	5.8	2	Coiling	Complete occlusion	No	0	12	Complete occlusion	0
5	F/58	SAH	1	L PCA: P2	Dissecting/ Saccular	6	3.8	Coiling	incomplete occlusion	No	1	6	Recurrence	0
6	M/47	SAH	2	L PICA: telovelotonsillar	Dissecting/ Fusiform	4.8	NA	PAO with coil	Complete occlusion	No	0	6	Complete occlusion	0
7	F/58	SAH	2	L SCA: anterior pontomesencephalic segment	True/ Saccular	3.4	2.4	Coiling	Complete occlusion	No	0	12	Complete occlusion	0
8	M/27	SAH	2	PICA: lateral medullary	True/ Saccular	4	2.8	Coiling	Complete occlusion	No	0	12	Complete occlusion	0
9	M/31	SAH	2	L PCA: P2	Dissecting/ Saccular	4.2	2.8	Coiling	Neck remnant	No	0	6	Recurrence	0

10	F/26	SAH, parasellar hematoma	4	L PCA: P2	Dissecting/ Saccular	12	2.4	PAO：coil	Complete occlusion	CI: temporal occipital lobe	5	6	Complete occlusion	4
11	M/58	SAH	2	R AICA：premeatal	Dissecting/ Saccular	3	1.8	PAO：Onyx	Complete occlusion	CI: Right cerebellar	1	9	Complete occlusion	1
12	F/66	SAH+intraventricular hemorrhage	3	R AICA：Meatal segment of Lateral pontine	Dissecting/ Saccular	3	1.8	Proximal PAO：coil	Complete occlusion	No	1	9	Complete occlusion	0
13	M/63	SAH	3	L PICA, branch of lateral medullary	Dissecting/ irregular	2	2	PAO：Glue	Complete occlusion	CI: Cerebellar and medulla	3	6	Complete occlusion	2
14	F/32	SAH	2	R PCA，P2	Dissecting/ Fusiform	16	NA	PAO: coil and glue	Complete occlusion	CI: temporal occipital lobe	1	6	Complete occlusion	1
15	M/56	Asymptomatic	0	L PICA, lateral medullary	Dissecting/ Saccular	2	1.5	SAC	Complete occlusion	No	0	9	Complete occlusion	0
16	F/57	SAH	2	L PICA: cortical segment	Dissecting/ Saccular	7.7	2.5	PAO:Onyx	Complete occlusion	No	0	12	Complete occlusion	0
17	M/60	SAH	2	L PICA, telovelotonsillar	Dissecting /irregular	3.5	NA	PAO: Onyx	Complete occlusion	No	1	6	Complete occlusion	0
18	F/68	SAH	2	R AICA, Meatal segment of Lateral pontine	Dissecting /irregular	3.2	NA	PAO:Onyx	Complete occlusion	No	2	6	Complete occlusion	1
19	M/68	Asymptomatic	0	R PCA, P1-2 junction	Dissecting /Saccular	3	2	SAC	Complete occlusion	No	0	9	Complete occlusion	0

Abbreviations:SAH, subarachnoid hemorrhage; M, male; F, female; L, left; R, right；PCA, posterior cerebral artery; PICA, posterior inferior cerebellar artery; AICA, anterior inferior cerebellar artery; SCA, superior cerebellar artery; PA, parent artery; HHS, hunt-hess scale; PAO, parent artery occlusion; SAC, stent-assisted coil embolization；CI, cerebral infarction; mRS, modified rankin scale.
